# A review of methods of age estimation based on postmortem computed tomography

**DOI:** 10.1093/fsr/owae036

**Published:** 2024-07-18

**Authors:** Marta Barszcz, Krzysztof Jerzy Woźniak

**Affiliations:** Department of Forensic Medicine, Jagiellonian University Medical College, Krakow, Poland; Doctoral School of Medical and Health Sciences, Jagiellonian University Medical College, Krakow, Poland; Department of Forensic Medicine, Jagiellonian University Medical College, Krakow, Poland

**Keywords:** age estimation, postmortem computed tomography, forensic imaging

## Abstract

Age at death is one of the key elements of the “biological profile” prepared when analysing unidentified human remains. Biological age is determined according to physiological indicators and developmental stage, which can be determined by bone assessment. It is worth remembering that the researcher must interpret each case individually and in accordance with the current state of knowledge. One of the most developed tools for analysing human remains is postmortem computed tomography. This allows for the visualization not only of bones without maceration but also of the entire body under various altered states, including corpses in advanced stages of decomposition and burnt bodies. The aim of this review is to present the current methods for age estimation based on postmortem computed tomography evaluation, comparing the results presented in 18 research projects published between 2013 and 2023 on foetuses, children, and adults from contemporary populations. Recent literature includes assessment of bones and characteristics such as skulls, teeth, vertebrae, pelvises, and long bones to estimate age at death. We cover the methods used in this recent literature, including machine learning, and discuss the advantages and disadvantages of them.

**Key points:**

- Postmortem computed tomography allows the analysis of several areas of the body at the same time, which may not be possible in the case of clinical trials (where the examination area should be limited).
- Postmortem computed tomography may enable the collection of data from people whose clinical examinations are relatively rare (e.g., pregnant women, children).
- Artificial intelligence should increasingly be used in studies on age estimation.
- Further research on modern populations is necessary to verify and refine the methods used to estimate age at death.

## Introduction

Age at the time of death is one of the key components of the “biological profile” compiled whenever unidentified human remains are being analysed [1, 2]. The age of an individual at the time of death may be interpreted as either chronological or biological (also known as physiological) age [2, 3]. Establishing the chronological age of a deceased person requires dates of both birth and death. In contrast, biological age is determined according to physiological markers and the stage of development, both of which can be determined by evaluating bones. From the moment a relationship is indicated between an individual’s biological and chronological age, the biological age can be used to establish the age of any unidentified human remains [1]. Nonetheless, it is important to note that biological age does not always correspond exactly with chronological age [3]. This potential discrepancy increases with age and is known as the trajectory effect [3]. Moreover, some areas of the skeleton that are used to establish biological age differ between the male and female sexes [2]. For example, significant differences between the sexes have been observed in the rate of ossification of the fourth costal cartilages [4–6].

The first attempts to organize the methods of establishing age at the time of death were undertaken by Ubelaker in 1987 [7]. Over time, other researchers have developed and refined the methods, adjusting them for specific populations. The most up-to-date compilation of age assessment methods was presented by Cunha, and includes both subadults (foetuses, newborns, infants and children, adolescents) and adults. Cunha’s paper also presents age assessment methods specifically designated for remains in specific conditions, such as decomposition, skeletonization, or fragmentation [8]. The work by Cunha was published in the year 2009, and was mainly devoted to conventional methods of anthropological examination, although it mentioned the use of clinical radiology. The advancement of publications based on post-mortem imaging studies at that time did not allow their inclusion.

### Radiology in forensic anthropology

The discovery of X-rays by Wilhelm Röntgen in 1895 opened new possibilities not only for clinical trials but also for forensic analyses, and X-rays began to be used for identification purposes in cases of civil and criminal law [9].

Advances in radiographic techniques led to the development of the first computed tomography (CT) scanner, patented in the year 1968 [10]. Initially, CT scanners were used for diagnostic purposes, but over time they also began to be used in physical anthropology, e.g. for examining mummies without the need to unswathe them. The first such analysis took place in 1976, when the mummified brain of a 14-year-old boy was scanned [9]. Currently, CT is used for bone measurements and nonmetric (qualitative) analyses intended for stature and sex determination. CT is convenient for these purposes because it requires neither prior bone preparation through maceration nor any particular bone positioning. The analysed remains may be positioned as found, which minimizes the risk of damage to them. This is particularly important in cases of burnt corpses [11]. Currently, postmortem computed tomography (PMCT) is increasingly being used in forensic pathology facilities as a tool to complement conventional postmortem examination [12–14].

PMCT examinations help to obtain data that can be analysed in many aspects. For instance, PMCT has recently been used for postmortem interval (PMI) estimation, which refers to the time expired from the time of death to the time when a body is found. As a result, the radiological alteration index (RA-index) was proposed, with this referring to the progress of postmortem changes [15]. It may often be useful to differentiate between early and late PMI. For this purpose, a radiomics analysis has been performed on PMCT images of liver and pancreas samples [16]. Radiomics is an approach to data processing that is based on obtaining quantitative data and processing information from diagnostic images that, under standard conditions, would be impossible to detect by the human eye. This enables better recognition of tissue shape changes and heterogeneities [17]. Klontzas et al. [16] found that machine learning (ML) models and combined radiomics data from the liver and pancreas were useful for PMI prediction.

Another concept that can be useful for PMI estimation is observation of postmortem hypostasis. Using PMCT, it is possible to observe hypostasis inside a human body. Zerbini et al. [18] conducted a prospective study investigating axial views of the heart and inferior pulmonary vein. PMCT images were repeatedly acquired over time, from the time death confirmed in the hospital. The results showed that the more measurements taken, the smaller was the standard error; if PMI was <12 h, it could be calculated with SD of <2 h. They observed stabilization of the density gradient at 12 h after death, a mechanism involved in the fixation of hypostasis.

### Subadults versus adults

Age assessment based on the changes that occur during growth and development is more precise and accurate than that based on degenerative changes [1].

Irrespective of the study method used, it is important to realize that chronological age is not always consistent with biological age, either dental or skeletal [2]. This is particularly noticeable and relevant in children, in whom various developmental changes occur within short time intervals [19].

According to Cunha, it is also important to remember that most methods also depend on sex and population affinity.

#### Subadults

The following age groups are distinguished within the subadult group: foetuses, newborns, infants (0–6-year-olds), children, and adolescents [8]. Age assessment in foetuses is most commonly based on the presence of primary and secondary ossification centres [20, 21] or the length of long-bone shafts [19, 22, 23]. X-rays are very useful for analysing foetal corpses and body parts. The analysis of dead newborns is performed using similar methods, and additionally, according to the state of the fontanels [8]. Age analysis based on tooth development is most useful in the case of foetuses, newborns, infants, and children [7, 24–27]. Tooth-based age assessment methods have been reported to be the least susceptible to environmental factors, unlike methods based on assessing other parts of the skeleton [19]. Age assessment in adolescents is based primarily on tooth development [24, 28] and analysis of hand and wrist bones with the use of X-ray analysis and tools such as the Greulich and Pyle atlas [29] and the Tanner–Whitehouse score [30, 31].

Age estimation at the time of death is mostly based on the development of primary and secondary centres of ossification [20, 21], epiphyseal closure [19], and tooth calcification and eruption [24, 28]. Because these topics have already been well researched, new methods of PMCT-based age estimation are uncommon. PMCT is usually used as a tool for determining the cause of death [32, 33] or establishing guidelines for specialists in forensic radiology [34].

#### Adults

Physical growth stops between the age of 20 and 25, which marks the end of “adolescence” and the beginning of the “adult” phase, and is characterized by obliteration of the spheno-occipital or basilar synchondrosis [35, 36] and fusion of the sternal end of the clavicle [37–39]. In adults, conventional age analysis is based on assessing the entire skeleton for signs of degeneration [8], as well as assessing the pubic symphysis [40–43], auricular surface of the pelvis [44], sternal end of the fourth rib [4, 45], and cranial sutures [46–49]. Over time, age assessment methods have been tested, improved, and adjusted for specific populations [1]. However, such methods as bone histology [50, 51] are less common and may be inaccessible in some research centres because they require specialized equipment [1].

### Body regions included in PMCT articles

#### Skull

The use of cranial sutures for estimation of the age at the time of death goes back to the 1920s [46]. Since then, researchers have improved and critically reviewed the methods based on ossification of these bone junctions. Ultimately, cranial suture closure is not believed to be reliable for age estimation because no statistically significant correlation has been found between the individual’s age and the degree of suture obliteration [52–54].

Tooth analysis is the basis of many age estimation methods for human remains [55, 56], although advanced decomposition may lead to tooth detachment and loss [57].

#### Pelvis

Most of the previous studies that we found that estimated the age at the time of death in adult subjects were based on the pelvis. Analysis of pelvic bones may be challenging because of the abundance of soft tissues. Nonetheless, despite some blurring, trabecular structures can be seen clearly enough on CT scans [58]. The most commonly used methods for age estimation based on the pelvic region are the auricular surface phase method by Lovejoy et al. [44] and the pubic symphysis method described by Brooks and Suchey in 1990 [43]. In the Australian population, the Suchey-Brooks method was better suited to female than to male individuals. The sixth phase, which is characteristic of the oldest individuals and manifests as porosity and irregularity of the pubic symphysis surface, was identified correctly in all relevant subjects from the study population [58]. However, despite promising results in female individuals and in phase six subjects, the Suchey–Brooks method should not be used as a reliable and definitive tool for age estimation in men or women of the Australian population [59].

Another method for age estimation is that based on the acetabulum by the Rougé-Maillart team. This method is based on degenerative changes on the acetabular rim, the acetabular fossa, and the absence of activity of the posterior horn [60].

#### Other body regions

Fusion of the medial clavicular epiphysis is complete in all individuals at the age of 29 [19]. In older individuals, age estimation can be based on degenerative changes [8] in areas such as the joints and sites of attachment between bones and cartilage. According to the Centers for Disease Control (CDC), osteoarthritis is the most common degenerative joint disease; it progresses slowly with age and affects mostly women over 50 years of age [61]. Osteoarthritis affects not only limb joints but also the spine, where in extreme cases it may lead to spinal stiffness through vertebral fusion. This fact has been used by numerous authors who evaluated various parameters of bone juncture sites.

Another area of the skeleton that may be analysed for age estimation involves the thoracic and lumbar vertebrae, where observable age-related degenerative changes in vertebral bodies occur earlier and are more common than in other areas of the skeleton [62]. These changes include the development of osteophytes, defined by van der Kraan as “fibrocartilage-capped bony outgrowths” [63]. Such changes are reported to show a significant correlation with age [64–69].

The cartilage changes observed with age manifest as calcification or loss of thickness, which may lead to osteoarthritis [70]. Anthropologic analysis of skeletons is based on observable changes to cartilage, particularly costal cartilage, with İşcan’s method recommended for analysis of the thorax [8]. Another region of cartilage changes involves the thyroid cartilage, where morphological changes, including cartilage calcification, can be observed *via* X-ray with the methods described by Turk and Hogg [71] and Garvin [72]. However, age-related changes in cartilage morphology are characterized by high individual variation [73].

#### QCT and ML

Quantitative computed tomography (QCT) is a tool used to calculate bone mineral density (BMD). In the case of QCT, it is necessary to use phantom scanning, which may allow a correlation between Hounsfield units (HU) and the measurable quantity to be found [74]. This involves placing a phantom made of solid calcium hydroxyapatite under the patient’s bone, most often the hip bone or lumbar spine. When analysing the QCT image, a linear regression is applied between the mean HU values of the areas of interest and the known concentrations of bone equivalent material. The result is then used to calculate the average HU per concentration (given in mg/cm^3^, i.e., bone mass per unit volume of tissue) of bone equivalent material in the region of interest. Some researchers found the results of QCT to be better than those of another popular tool, dual-energy X-ray absorptiometry (DXA) [75].

ML, which is an area of artificial intelligence (AI), seems to be the future of numerous scientific disciplines [76], including the forensic sciences, where we can find increasing use of ML, particularly for age estimation in subadults. Some of the first studies in which ML was used were based on X-ray and MRI images of the hand [77–84], X-ray images of the pelvis [85], and MRI images of the knee [86]. Imaging of the hand is the fastest way to obtain information about age from X-rays of living patients. The development of bones is clear, and there is very little X-ray exposure to inner organs, especially the gonads and the thyroid gland. At the other extreme is imaging and analysis of the pelvis, where research might be difficult for non-experts, and the inner organs are exposed to ionizing radiation. Knee joints may be particularly problematic for undertrained interpreters.

## Materials and methods

The purpose of this study was to present and summarize age assessment methods using PMCT imaging.

The data for this study were obtained using the PubMed and Google Scholar search engines.

The following keywords were used for the PubMed search engine: age estimation; PMCT “or” postmortem computed tomography “and” age. The keywords used for the Google Scholar search were: age estimation; PMCT “or” postmortem computed tomography. Additional inclusion criteria were: English language, original article, the chronological age of subjects was known, research was performed on human PMCT, and appropriate scientific organization of the article. Duplicates from the databases were counted only once.

The initial PubMed search yielded 202 articles. After the timeframe was limited to the years 2013–2023, the number of articles decreased to 130. Eventually, we selected nine articles that best fitted the topic of this paper (Table 1).

**Table 1:** First authors whose works were used for this review.

Authors	Year of publication	Number of subjects
Chiba et al. [87]	2013	125
Sathapana et al. [88]	2013	82
Villa et al. [58]	2013	319
Brough et al. [89]	2014	19
Chiba et al. [90]	2014	199
Ishii et al. [91]	2016	121
Aramaki et al. [92]	2017	131
Savall et al. [93]	2018	Reference sample 1 100; test sample 75
Hall et al. [59]	2019	204
Monum et al. [94]	2020	320
Feld et al. [95]	2020	13
Belghith et al. [96]	2021	200
Barszcz et al. [97]	2021	255
Pham et al. [98]	2021	814
Imaizumi et al. [99]	2021	707
Chiba et al. [100]	2022	250
Indo et al. [101]	2022	25
Kondou et al. [57]	2022	277

The initial Google Scholar search yielded 2 910 articles. Once the timeframe was limited to the most recent decade, 2 300 articles remained. Finally, nine additional articles were ultimately selected (Table 1).

## Results

### Subadults

Calcification of the clavicle and deciduous and permanent teeth was used by Indo et al. to analyse 25 foetuses between gestational weeks 13 and 33 [101]. Their study showed a good correlation between the estimated foetal age and calcification of all deciduous teeth (*r* = 0.87 – 0.98). In addition, calcification of the clavicle appeared earlier than calcification of the deciduous teeth; the calcification rate of bone was slower than that of deciduous teeth. The authors concluded that calcification of bone may be effective for age estimation under a gestational age of 19 weeks.

The group of foetuses analysed by Feld et al. [95] was smaller and included 13 bodies. The authors assessed the clavicle, femoral length, and femoral bone nuclei, and compared their findings with other collected parameters. They observed a discrepancy (maximum of 36 d ±18.64 d) between the individual assessment methods, with the mean deviation being dependent on the analysed elements. The biggest mean deviation was observed when clavicle length and distal femoral epiphysis were compared (1.71 weeks), and the smallest when clavicle and femoral length were compared (6.82 d). Gestational age assessment based on clavicle length yielded a younger age in comparison with age assessment based on femoral length. The study also showed a discrepancy in the estimated gestational ages based on femoral length and the diameter of the distal femur.

Dentition in children was the next element to be evaluated for age estimation. PMCT of 19 maxillae and mandibles was compared with conventional orthopantomography (OPT) [89]. Age was known to range from 0 to 18 years. Three independent observers conducted dentition-based age estimation separately for data obtained from OPT “virtual constructions” and PMCT scans. To increase the reliability of the study, dental age was assessed with the Demirjian and Al Qahtani methods. Statistical analysis showed no significant difference between the predicted ages for the two tools for each rater. One additional advantage of this study was the fact that the results were repeatable, despite the raters’ diverse professional backgrounds: a forensic anthropologist with PMCT experience, and dental practitioners with dental age estimation experience. PMCT has an advantage over OPT in that it facilitates the possibility to isolate a single cross section that can be analysed in detail.

### Adults

#### Skull

Analysis of the sagittal suture for age estimation at the time of death in the modern population remains questionable. The two main studies that used this method differed in the way the authors divided the suture into segments, the scale of suture closure, and the study group characteristics (Table 2).

**Table 2:** Comparison of the assessment methods used in the studies by Barszcz and Woźniak [97] and Chiba et al. [87].

Characteristics	Study group	
	Barszcz and Woźniak [97]	Chiba et al. [87]
Group characteristics	Polish population: 255 males	Japanese population: 125 individuals; 64 males; 61 females
Suture segments	Four segments identified based on the shape of the suture—analysis of the entire length of the suture and its sagittal sections	Four segments—a 10 mm block cut out of each segment—with a stack of 20 cross-sectional images obtained from each segment analysed
Type of analysis	Volume-rendered and multiplanar reconstructions	Volume-rendered and multiplanar reconstructions
Type of scale	For multiplanar reconstruction: indentation “notch” 0–4; suture variations 0–7 For volume-rendered reconstruction: 0–6	For multiplanar reconstruction: 0–6 Three methods: frequency, average, and maximum method

Chiba and colleagues, who used the “average method” (unexplained term), suggested that cranial suture obliteration occurs faster in females. Three regression equations were derived to determine age at the time of death. The standard deviation was lower in females (±29.6 years) than in males (±33.6 years), with the value for both sexes combined being ±31.4 years. The authors reported that combined with other methods, PMCT may be useful for estimating age at the time of death, particularly in adult women and in cases where a skull is the only recovered piece of human remains [87].

Multiplanar reconstructions and volume-rendered reconstructions were used by Barszcz and Woźniak in their study, in which they indicated that sagittal suture closure in Polish males tends to begin at the inner surface of the skull, a tendency that is true for all segments of the suture. These findings may be used in further age estimation studies. Nonetheless, the authors emphasize that this method is associated with a high risk of error, of up to 17.46 years on the inner table of the skull and up to 17.41 years on the outer table [97].

Another study assessed possible age-related changes in alveolar bone cortical thickness (ABCT) in the maxilla and mandible, which is a key parameter for the implantation of temporary anchorage devices such as miniscrews. A total of 82 PMCT scans (half female and half male subjects) were analysed [88]. The only statistically significant difference in ABCT between the sexes was shown at the buccal side of the maxillary molar region, where the mean thickness was greater in women than in men. Correlations between ABCT and age were shown in the maxillary incisor region on the labial side, in the maxillary incisor and premolar regions on the palatal side, and in the mandibular incisor region on the labial side. The only location that showed a statistically significant negative correlation was the mandibular canine region. Moreover, the ABCT at the lingual side of the retromolar region showed a statistically significant difference between the sexes, with the thickness being greater in women than in men. However, all measurements that showed a statistically significant difference were so small that their clinical relevance was considered negligible.

The mental foramen was the next region of interest used for age estimation at the time of death, being analysed by a team of Japanese researchers led by Ishii et al. [91]. A total of 121 Japanese cadavers of known age and sex were subjected to PMCT analysis. To ensure consistent measurements, the mandibular plane of each skull was first defined and identified, and then the perpendicular “coronal” and “transverse” planes were defined to observe and measure the mental foramen opening direction, with two parameters of this being measured on each plane. The study showed no significant relationship between age and either the θ1 angle (*P* = 0.709) or the θ2 angle (*P* = 0.392) in women. However, in men, the θ1 angle measurements yielded significant age-related differences in the coronal plane. The derived regression equation explained the changes in angle θ1 with age. Namely, angle θ1 increased until subjects reached their early 50s and thereafter decreased with age. Angle θ2 showed a significant (*P* = 0.002) linear correlation with age. The regression equation for this angle showed the decrease with age. The authors concluded that although males show age-related changes in the mental foramen opening direction, females do not.

#### Pelvis

Evaluations of axial views of the pubic bone, using dense and homogeneous bones for multiplanar reconstruction, showed that both the left and right side of the pubic symphysis helped identify four phases, including a juvenile trait [58]. The authors also evaluated the auricular surface of the ilium at the level of the first and second sacral vertebrae, which helped identify five phases, including a senile trait. Because pubic bone phases 2 and 4 and auricular surface phases 4 and 5 showed sex-dependent differences, the data from female and male subjects were analysed separately. Both evaluated regions showed a positive phase-age correlation, which was more pronounced in women than in men. This indicates that bone density decreases with age because higher phase numbers mean lower bone density. The strongest correlations of 0.91 (*P* < 0.001 in women) and 0.78 (*P* < 0.001 in men) were observed when the results from the pubic bone and the auricular surface were combined for analysis. Importantly, the authors set the upper age limits for age estimation at 80.5 ± 7.7 years for women and 71.7 ± 11.0 years for men, consistent with the fact that male subjects showed a weaker correlation.

The pubic symphysis can be measured at 10 distinct sites in the axial and coronal planes. Depending on the specific measurement site, it can demonstrate positive and negative correlations with age [90]. One parameter, the pubic bone length of the parallel division of the anterior–posterior direction, showed considerable differences between the sexes; therefore, the presented results were stratified by sex. A negative correlation was observed between the soft-tissue thickness measurements at the pubic symphysis and age, indicating a tendency for tissue thickness reduction with age. Therefore, this method, in conjunction with other methods used on the same skeleton, may be a useful tool for age estimation at death.

Savall et al. [93] used only left-side measurements in their study. They observed the greatest inaccuracy and bias in males aged 71–100 years and in females aged 56–70 years. The overall conclusion drawn from their study was that age estimation tends to be much less accurate in the elderly, resulting in underestimation of age in such individuals. The authors emphasized that in the case of cadaveric bones from decomposed or burnt remains, evaluation against a three-dimensional model obtained *via* CT would be more appropriate than the Suchey–Brooks method based on dry bones. Despite their promising results, the authors were reluctant to recommend the evaluated method for age estimation in the French population, advising caution in its use and stating the requirement for further expansion of the reference sample, specifically by including more female subjects.

PMCT scans of 200 (predominantly left-sided, with 29 right-sided cases) acetabula were used in one study [96]. Three-dimensional volume rendering was performed, followed by the global illumination rendering technique, which produces more photorealistic images. The assessment procedure was based on the Rougé-Maillart method, and included the acetabular rim (stages 1–5), apical activity of the posterior horn (stages 1–4), and acetabular fossa (stages 1–4). The summative scores for the individual aspects of each analysed acetabulum were calculated, and statistical analyses were conducted, including a box plot of age with use of the criteria of the Rougé-Maillart method. The authors developed several regression models, with models No. 3 (using the stages based on evaluating the acetabular rim, apical activity of the posterior horn, and the acetabular fossa) and No. 4 (based on the total score of all stages) being the most accurate. A morphological analysis showed the acetabular rim to be the best age marker. In contrast to the acetabular rim, the apical activity of the posterior horn showed the lowest correlation with age. The authors concluded that this was a promising method of age estimation at the time of death, with a prediction rate of 85.6% based on morphology criteria.

#### Other techniques

Monum et al. [94] used volume rendering reconstructions to evaluate the sternum, xiphoid process, costal cartilage, and rib ends. These areas were isolated and assigned the following scores depending on the stage of ossification: 1—ossification of the first costal cartilage (OF), 2—ossification of the second to seventh costal cartilages at the rib ends (OR), 3—ossification of the second to seventh costal cartilages at the sternal ends (OS), 4—fusion of the manubriosternal joint (FM), and 5—fusion of the xiphisternal joint (FX). The summative scores for the individual areas were used in statistical analysis. Most analysed areas (except for FM) showed a significant correlation with age in both sexes. The highest score for both sexes was observed at the left OS (male: *r* = 0.60, female: *r* = 0.65). Unfortunately, the appearance of ossification alone does not seem to be a good marker of minimum age, although it may play a role in limiting the age range to be considered for the individual in question. A lack of discernible ossification was observed at FX, FM, and OR in individuals up to 50 years of age, whereas OS and OF showed no discernible ossification before the ages of 30 in men and 40 in women. Significant differences between the sexes were observed in the evaluated areas, except for OR on the right side. Therefore, the authors concluded that the methods did not present a reliable marker for age estimation in the Japanese population.

Evaluation of thoracic and lumbar vertebrae and their osteophytes using a bone kernel reconstruction allowed development of a methodology for individual vertebra types: the length of the most prominent osteophyte in a plane parallel to the upper (or lower) vertebral body planes was measured using a scale from 0 to 5 (“measurement O”), with the score O (from 0 to 5) reflecting osteophyte formation [100]. Sagittal and coronal sections were used to evaluate the fusion between vertebral osteophytes, with score B (from 0 to 2) reflecting osteophyte fusion. Statistical analysis showed no significant differences between the sexes. Subsequent findings were related to osteophyte measurements and descriptions. For example, if O(max) was 5, the regression equation yielded an estimated age range of 63.6–83.2 years in women and 59.7–79.9 years in men. Seven regression equations were created based on the main variables; however, only one variable, the number of vertebrae with clear osteophyte formation (O ≥ 2), showed a standard error of the estimate of <10 for both sexes. Therefore, this parameter was considered to be effective. Receiver operating characteristic curve analysis was also used and provided additional valuable information.

The next new methodology developed analysed 131 male thyroid cartilages using a qualitative scale to evaluate the stage of cartilage calcification at six areas: the superior horn, cranial branch, midline, paramedian bar, caudal branch, and spongy change in the inferior horn [92]. The Kruskal–Wallis test yielded statistically significant differences in calcification rates between those in the 20s and 50s and those in the 30s and 50s. There were also changes in the qualitative analysis; e.g., no individuals under the age of 40 had a score of 6 for both superior horns, and a score of 3 for the paramedian bar first occurred at an age of ~30 years. Nonetheless, the findings also showed that although the mean calcification rate and the mean CT density increased with age, this process stopped after the fifth decade of life. In addition, statistical analyses showed no significant differences between most of the analysed age groups, making this method unreliable for forensic purposes. However, the authors found evidence of a reduction in the calcified area (i.e. regression of calcification changes) in some older age groups.

#### QCT and ML

It was reported that as decomposition progresses, vertebral bodies begin to show the presence of gas, which interferes with BMD interpretation. Therefore, the Vertebral Putrefaction (VP) Grading System (from 0 to 5) was introduced. This system allows vertebra with a VP grade of 3 or higher to be excluded from further analysis [57]. QCT and BMD focused on vertebra T10 were used as tools for age estimation. The study showed that the original (CT-based) BMD value had a strong relationship with the approximate (calculation-based) BMDs, and provided evidence that the VP grade increases with the amount of time elapsed from the time of death to corpse recovery. Moreover, a negative correlation was found between estimated BMD and age at death, in both women and in men. The authors concluded that this method may be helpful for estimating age at the time of death.

Among the papers published within the analysed time interval, there were two studies that used ML, one by Pham et al. [98] and the other by Imaizumi et al. [99]. The first study included 814 corpses whose femurs and mandibles were analysed after images of these bones were extracted from whole-body PMCT scans. Three-dimensional voxels were subsequently fed into a convolutional neural network (CNN) model. Age estimation was defined as a regression problem. One of the main challenges faced by the authors was to teach the network how to interpret the data that it was being fed. The authors also had to assess which of the models that they used was associated with the lowest mean absolute error (MAE). Ultimately, they evaluated three CNN architectures with their dataset: (i) mandibles and femurs were considered separately (MAE of 7.07 ± 0.51 years and 5.74 ± 0.04 years, respectively); (ii) mandibles and femurs were combined to obtain late fusion and early fusion data (MAE of 5.41 ± 0.26 years and 5.46 ± 0.37 years, respectively); and (iii) mandibles and femurs were combined in a single model using the middle fusion technique. The algorithm used by Pham et al. provided age estimation within <1 min. This algorithm worked best with combined data for the femur and the mandible and the use of middle modality fusion, yielding results that were within acceptable limits for forensic age estimation (< ± 10 years).

Imaizumi et al. [99] evaluated both male (*n* = 617) and female (*n* = 190) cadavers. They analysed the superior half of the right femur, the vertebral body of the third lumbar vertebra, the ischial tuberosity, and the iliac crest of the right coxal bone. Following volume rendered reconstruction, images of these selected bones were exported and stored in TIFF format. Regions for further analysis were selected and clipped by placing landmarks in certain anatomical locations, which were consistent for each individual. Further processing resulted in areas to be analysed that were homologous in shape and whose pixels corresponded to the same anatomical locations in all subjects [95]. Two methods, principal component analysis and partial least squares regression, were used to reduce dimensionality. The resulting estimated ages of vertebral bodies were consistent with actual age in both sexes. MAEs were calculated for each of the evaluated bone areas: in males, the MAEs were higher in the age groups at the extreme ends of the analysed age range (i.e. in those aged 20–30 years and 80–90 years) than in mid-range age groups (40–70 years). In females, high MAEs were only observed in the youngest age group (20–30 years). The most accurate estimation was achieved in vertebral bodies in males and in the ischial tuberosity in females [99].

## Discussion and conclusions

In line with earlier reports on the possible use of PMCT, Feld et al. [95] and Indo et al. [101] focused on evaluating dead foetuses to estimate gestational age at death as early as week 13 of gestation. This is the time when initial calcification of the teeth is detectable in PMCT images [101]. These findings were contested by Kraus, who did not observe such findings. The study by Kraus included the use of histological staining, detection of calcification, and analysis with the use of a stereoscopic microscope with a 15× eyepiece [102]. Notably, these studies were conducted 63 years apart and used different methodologies.

Differences between the right and left side of the pubic symphysis were found in more than one-fourth (27%) of the evaluated subjects [59]. This is an important finding because it may affect the final age estimate if only one side of the pubic symphysis is available for analysis. The same conclusion was reported by Lottering et al. [103]. Savall et al. [93] were of the same opinion, and emphasized that the phenomenon of asymmetry had been reported earlier [104].

Discrepant results in studies conducted within the same populations were also addressed by Belghith et al. [96], who suggested that the discrepancies may be due to potential differences in the socioeconomic status of some subgroups of subjects within the study population, with the population as a whole still being classified as “middle class”. Study results may also be affected by the predominance of one sex in the study population. Notably, Hall et al. [59] and Savall et al. [93] all reported their methods to be insufficiently reliable for age estimation in older women [59, 93]. This may be due to the fact that postmenopausal women experience hormonal changes that accelerate bone degeneration and osteoporosis [105, 106], potentially leading to inaccurate interpretation of pubic symphysis structure. However, the authors of these studies overcame this problem.

Sathapana’s et al. paper [88] is an excellent example of the fact that even papers that are not strictly categorized as forensic literature can have forensic applications. This confirms the need for interdisciplinary studies and the use of unconventional solutions to research problems. The body of knowledge available from other disciplines may also be used in studies in the forensic sciences*.* One example of this may be the osteoporotic changes that develop with age and are characterized by bone mass reduction progressing at an abnormal rate. Importantly, bone mass reduction is also a process that occurs spontaneously over time [70, 107]. Such changes in age estimation at the time of death were used by Kondou et al. [57], which was important because their study included both male and female subjects.

AI may be increasingly used in studies on age estimation in both living and deceased subjects. However, there are currently no standardized databases or methods on which deep learning can be based. An increasing number of studies and creation and expansion of databases may lead to more accurate and rapid age estimation based on PMCT-derived data and other data. Pham et al. [98] highlighted the importance of study sample size; the larger the sample size, the larger the pool of data available for ML, which translates into more accurate study results. The condition of the evaluated bones may pose a challenge for data acquisition. Advanced corpse decomposition or mummification may preclude skeletal assessments [15], while another obstacle may be due to the bone being too thin to be isolated from the surrounding soft tissues [99].

The authors of the studies cited above support the view that PMCT, which is gradually becoming the new standard in forensic anthropology [96], is an excellent modality for age estimation studies based on cadaveric material. Soft tissues do not obscure bone structure, and even the trabecular architecture can be observed [58,108], which considerably expands the scope of assessment, eliminating the need to focus solely on the presence or absence of a specific bony structure [19, 24, 28]. Because of this, PMCT offers the possibility to analyse the changes in the quality of this bony structure that occur with age. The use of additional volume rendering applications, such as the global illumination rendering technique, can help to obtain photorealistic images that can be easily interpreted, even by individuals with a limited background in radiology [96].
